# Field evaluation of female- and male-targeted traps for *Ceratitis capitata* (Diptera: Tephritidae)

**DOI:** 10.1093/jee/toae139

**Published:** 2024-06-27

**Authors:** Elliot T Howse, Rieks D van Klinken, Nicholas J Beeton, Helen Spafford, Kim P James, Matthew P Hill

**Affiliations:** Department of Primary Industries and Regional Development, South Perth, WA 6151, Australia; CSIRO Health and Biosecurity, Brisbane, QLD 4001, Australia; CSIRO Data61, Battery Point, TAS 7004, Australia; Department of Primary Industries and Regional Development, South Perth, WA 6151, Australia; Department of Primary Industries and Regional Development, South Perth, WA 6151, Australia; CSIRO Health and Biosecurity, Black Mountain, ACT 2601, Australia

**Keywords:** monitoring, surveillance, attractant, ecology andand population dynamics, horticultural entomology

## Abstract

Mediterranean fruit fly (Medfly) *Ceratitis capitata* (Wiedemann) (Diptera: Tephritidae) is a globally significant economic pest for which lure based trapping can be used to monitor established populations and for surveillance. Either female- or male-targeted traps can be used; however, recommendations on which to apply are inconsistent and many programs rely on male-targeted traps. Here, we compare the performance of male-targeted traps (Lynfield Trap with Trimedlure) and female-targeted traps (Biotrap Globe trap with the 3-component lure—TMA Plus) in apple orchards in south-west Western Australia over 2 years (September 2019 to September 2021). Male-targeted traps caught more Medflies overall than female-targeted traps, although the difference was minor. However, female-targeted traps were better at attracting Medfly early in the season when populations were small; and were more likely to capture at least one fly when their paired male-targeted trap caught none. Conversely, male-targeted traps were more likely to capture Medflies late in the season and were more likely to catch high numbers of Medflies. Consequently, female-targeted traps may be better at detecting Medfly early in the season, and male-targeted traps may be better at detecting Medfly abundance late in the season, at least in apple orchards. Our results suggest that either or both trap-types could be used for monitoring Medfly populations, with the optimal solution being dependent on the intended application.

## Introduction

Mediterranean fruit fly (Medfly), *Ceratitis capitata* (Wiedemann) (Diptera: Tephritidae) is a global pest of economic and quarantine concern (Liquido et al. 1990, Malacrida et al. 2007). It is highly polyphagous, infesting more than 300 species of fruits and vegetables worldwide (Liquido et al. 1990). Trapping is used to demonstrate area freedom, and inform orchard management decisions and when used in high densities as a form of management (Jessup et al. 2007, Navarro-Llopis and Vacas 2014, Tan et al. 2014). Lures used in Medfly traps rely on attractants that are targeted towards either female or male Medflies (Shelly et al. 2014). Trapping is required to support pest-free area claims, or to demonstrate that population numbers have been managed and kept below critical thresholds (Lance and Gates 1994, Jang et al. 2014). This supports the trade of fruits and vegetables that are Medfly hosts.

Although some studies recommend the use of both female- and male-targeted trapping of Medfly (Epsky et al. 1999, Broughton and Rahman 2017), monitoring is typically conducted using male-targeted trapping systems (Hill 1987, Lance and Gates 1994, Wijesuriya and De Lima 1995, Epsky et al. 1999, Smith 2000, Broughton and De Lima 2002), using either, Capilure (CPL) or trimedlure (TML) (ICTA, California, USA). Female-targeted traps are used as a supplementary trapping system or in mass trapping as a control measure (Gilbert et al. 2013, Navarro-Llopis and Vacas 2014). Several female attractants with varying efficacy are available, with three-component BioLure used as the standard by Broughton and Rahman (2017) and Holler et al. (2009).

Several studies directly compared female- and male-targeted Medfly traps, Sciaretta et al. (2018) conducted trials between 2011 and 2013 on a single peach orchard in central Italy (near Rome) with traps set from Jun to Dec (Summer and Autumn) each year. They found that female-targeted traps caught Medflies earlier than male-targeted traps. Papadopoulos et al. (2001) conducted trials on a single mixed pome and stone fruit orchard in Northern Greece from Apr–Aug for a single season (Spring and Summer) and found that female-targeted traps caught Medflies earlier than male-targeted traps. Katsoyannos et al. (1999) found no significant difference in trap capture between female- and male-targeted traps, however, they did not directly assess the difference in trap captures across time and the study was only conducted over 2 months. Broughton and De Lima (2002) set traps in 2 citrus orchards in Bindoon, Western Australia from April 1999 (Autumn) to September 2001 (Spring). In this one locality, the authors found that male-targeted traps caught more Medflies, more often than the female-targeted traps, but did not comment on the seasonality of trap captures. Furthermore, Manrakhan et al. (2016) found that in citrus orchards in South Africa, male-targeted traps caught Medflies earlier than female-targeted traps.

We compared the performance of female- and male-targeted traps in and nearby apple (*Malus domestica*) orchards across 3 growing localities in southwestern Western Australia. We specifically compared female-targeted Biotrap Version 2 X globe traps containing the 3-component lure (TMA plus), to male-targeted Lynfield traps, containing trimedlure, across 2 complete years starting from apple budburst. Each of the 3 localities had differing Medfly abundance and host phenology. Examining the efficacy of the traps under these different conditions enabled us to evaluate the consistency of trap performance. We comment on whether female-targeted trapping alongside male-targeted trapping can provide a better resolution of seasonal population dynamics of Medfly in apple growing localities in Western Australia. Finally, we discuss how this information could be used to allow growers and regulators to make better, more informed management decisions.

## Materials and Methods

### Study System and Region

In Australia, Medfly was first detected between 1895 and 1897 in Perth, Western Australia (Sproule et al. 2001, Dominiak and Daniels 2012). Western Australia is the only state in Australia with an established population of Medfly. This population is genetically most similar to populations in Mediterranean Europe (Deschepper et al. 2021). Medfly is primarily present in the southwest of Western Australia (Woods et al. 2005, Dominiak and Daniels 2012), where it is a significant pest and affects a wide range of commodities including pome fruit, stone fruit, and citrus (Hancock et al. 2000, Liquido et al. 2017).

**Fig. 1.**
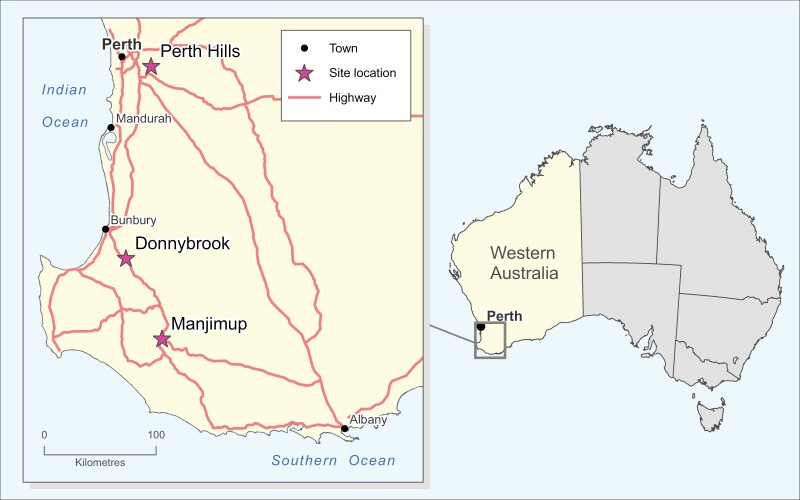
A map of southwest Western Australia, with stars indicating the location of trapping localities.

Trapping was conducted in 3 major apple growing localities in southwestern Western Australia: the Perth Hills, Donnybrook, and Manjimup (Fig. 1). These localities have a Mediterranean climate with hot, dry summers (December to February) and cool, wet winters (June to August). The northernmost locality (Perth Hills) experiences hotter summers and drier winters than the southernmost locality (Manjimup), which experiences milder summers and wetter winters (Belda et al. 2014). Medfly population dynamics differ between localities, partly due to their respective temperature differences, for example, De Lima (2007) reported that Medfly development was quicker in Donnybrook (warmer) than Manjimup (cooler). Duyck and Quilici (2002) found that there is a strong positive linear relationship between temperature and development rate for Medfly. Therefore, warmer climates may allow for more Medfly generations per year, resulting in greater potential abundance, though this is dependent on host availability and management practices.

Three available, commercial orchards were selected for trapping within each locality, apart from a 2.4 ha cherry orchard in Manjimup that was immediately adjacent to an apple block that we were not permitted to access, all traps were hung in apple blocks. However, orchards in the Perth Hills all grew both apples and stone fruit, one orchard in Donnybrook also grew cherries and other stone fruit, and one orchard in Manjimup also grew avocadoes. All orchards were also nearby to orchards growing cherries and other stone fruit. Property size in each locality was variable with sites in the Perth Hills ranging from (4.9–10.2ha), Donnybrook (6.4–54.0ha) to Manjimup (2.4–75.4ha). Apple cultivars grown across all localities varied from site to site. However, at all sites both early and late cultivars were grown on the same orchards. The period of harvest for apples in each locality varied slightly, with apples generally being harvested from the beginning of February to the end of April (Perth Hills), mid-February to early-May (Donnybrook), and late February to mid-May (Manjimup). Harvest time for cherries in Manjimup is mid-Dec to late-Jan), avocado harvest in Manjimup is (mid-Sep to Apr) and stone fruit harvest is (mid-Nov to late-Mar) in the Perth Hills and (late-Nov to early-Apr) in Donnybrook. Specific management practices were not recorded; however, each site was a commercial orchard employing standard management practices for the region.

### Trap Types and Placement

**Fig. 2.**
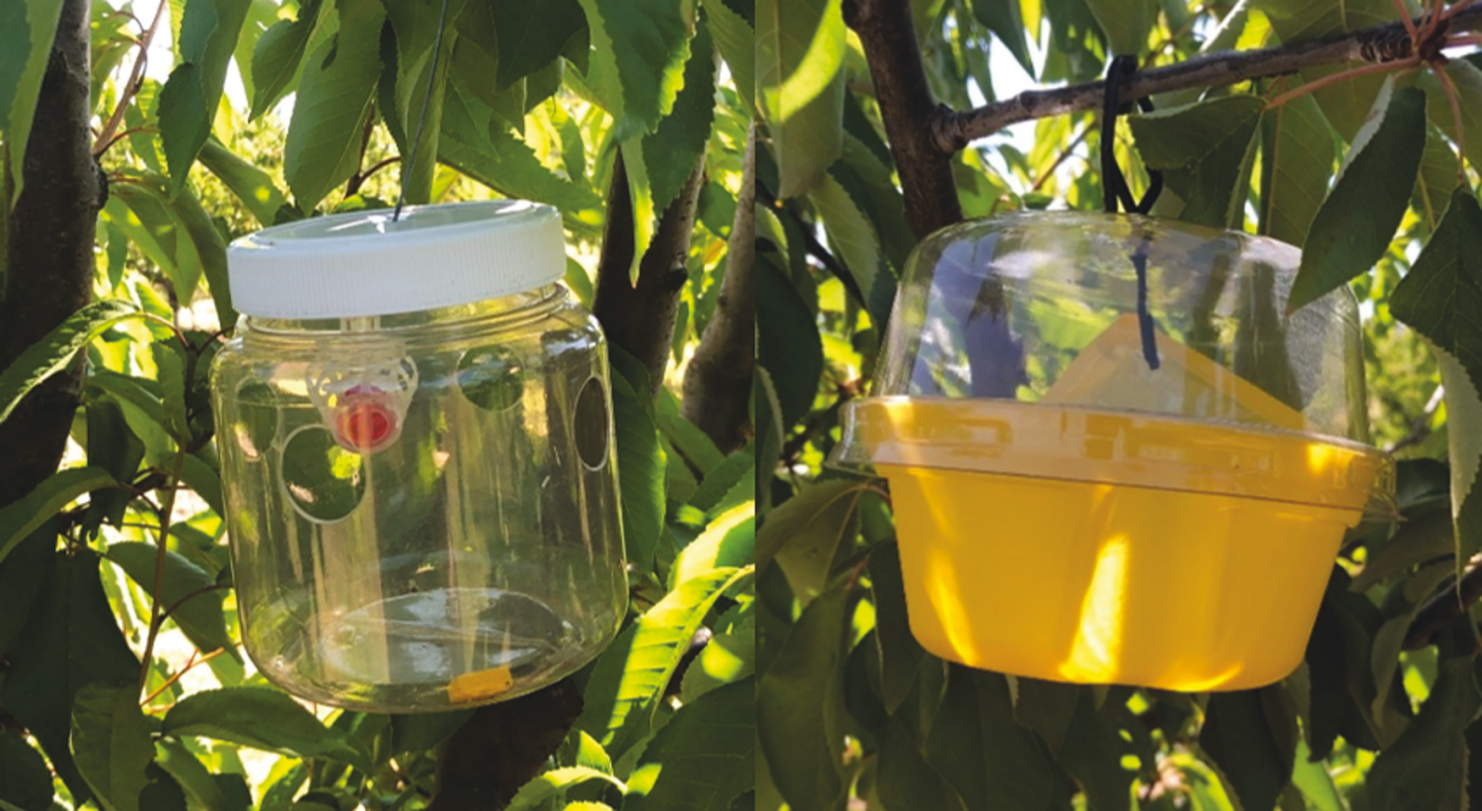
Male-targeted Lynfield trap baited with 3g trimedlure cone (left) and female-targeted Biotrap Version 2 X trap baited with TMA Plus Unipack—3 component lure (right).

Male-targeted traps consisted of a Lynfield trap (Biotrap, Victoria, Australia) baited with 3g trimedlure cone (Biotrap, Victoria, Australia) hung from the top of the trap in a clear plastic basket. The Lynfield trap consisted of a plastic 1-liter jar (12 cm diameter, 13 cm high) with an opaque white screw-on lid. Four entry holes 2.5 cm in diameter were placed 90° apart and 5 cm from the top of the trap (Fig. 2). A 1.47g dichlorvos cube (Biotrap, Victoria, Australia) was placed at the bottom as the killing agent.

Female-targeted traps consisted of a Biotrap V2 X Globe trap (Biotrap, Victoria, Australia) baited with TMA Plus Unipack, comprising ammonium acetate (36%), trimethylamine chloride (13%) and putrescine hydrochloride (6%) packaged in a 13 cm by 9 cm yellow sachet (Süsbin, Mendoza, Argentina). The Biotrap V2 X consisted of a 6 cm clear lid mounted on a 7 cm high yellow invaginated base with a 1 cm diameter hole in the center. The trap base had 4 semicircular invaginations equally spaced 90° around the perimeter, with a 1 cm diameter hole in the center. The total height was 11.5 cm and diameter at the junction of the base and lid was 15 cm (Fig. 2). A 1.47g dichlorvos cube (Biotrap, Victoria, Australia) was placed inside traps to kill any Medflies entering the trap.

Traps were set up in Sep 2019 in all 9 trial sites. Four pairs of female- and male-targeted traps were placed at each site. Traps were placed in a paired plot design. In 2019 they were placed in parallel rows of male-targeted and female-targeted traps, from Sep 2020 every second trap was swapped within each orchard for trap variability within rows. Traps in each pair were located at least 25 m from each other and other pairs. Where multiple apple cultivars were grown, paired traps were placed in the same cultivar. The traps were secured in the host tree canopy approximately 1.8 m from the ground. The traps remained in place year-round and were serviced monthly through to Sep 2021. On each trap-visit, the Medflies in each trap were removed, identified, counted, and sexed using a field magnifying glass, bycatch was not recorded. Trap lures and dichlorvos cubes were replaced every 3 months.

### Statistical analysis

To conduct analyses and allow for comparisons between localities, we first converted the number of Medflies caught to flies per trap per day (FTD). Unless otherwise specified, we inferred FTD from the trapping data using a Gamma-Poisson model with a Jeffreys prior, allowing for potentially different FTD rates between seasons, and simulating 100,000 times from the resulting posterior distribution. All analyses were performed using R statistical software (R Core Team 2020).

For all statistical analyses, we modeled the counts of the respective sexes and how these changed with respect to trap type and locality. To investigate overall differences between female- versus male-targeted trapping, we initially performed an analysis considering all orchards across all 3 localities. We also ran and compared the results of 2 binomial GLMs predicting the probability of the presence of Medflies in a site using one method (i.e., male-targeted or female-targeted respectively) by using the number of Medflies detected with the other method at that site as a predictor (i.e., female-targeted or male-targeted respectively).

Next, we looked at monthly analysis of female and male trap catches for all sites—a season is considered to run from Sep (when traps were set) until Sep the following year. In addition to trap type, we included the month of trapping in the analysis as a fixed variable, ignoring the effect of year, and compared the FTD between the trap types for each combination of site and month.

Finally, we investigated how the sex ratio changed between sites and over time (both month and year) using binomial GLMs and Tukey’s HSD post hoc test.

## Results

### Overall Performance

Total FTD across all times and locations was significantly different (*P* < 0.001, posterior comparison) between male-targeted (0.063 FTD) and female-targeted traps (0.053 FTD), though the size of the difference was relatively minor (Table 1).

**Table 1.** Total trap catches, percent of catch that are female, and trapping rate (flies per trap per day, FTD, and Bayesian 95% credible intervals) across all trap-visits in female-targeted and male-targeted traps in each locality and overall. Values for targeted flies are in bold. Letters show significant differences (*P* < 0.001) based on a Tukey HSD post hoc test between localities (for each trap type) and between trap types, for total, female, and male FTD.

Trap type	Locality	Total	% Females	FTD (flies per trap per day)		
				Total	Female	Male
Female-targeted	Perth Hills	96	86.5	0.011 (0.009, 0.013)a	**0.009 (0.008, 0.012)a**	0.001 (0.001, 0.003)a
	Donnybrook	995	83.1	0.113 (0.106, 0.120)c	**0.094 (0.087, 0.100)c**	0.019 (0.017, 0.022)c
	Manjimup	313	85.0	0.036 (0.032, 0.040)b	**0.030 (0.027, 0.034)b**	0.005 (0.004, 0.007)b
Male-targeted	Perth Hills	30	0.0	0.003 (0.002, 0.005)a	0.000 (0.0000, 0.0004)a	**0.003 (0.002, 0.005)a**
	Donnybrook	1368	6.1	0.156 (0.150, 0.166)c	0.010 (0.008, 0.012)c	**0.146 (0.140, 0.156)c**
	Manjimup	259	4.6	0.029 (0.026, 0.033)b	0.001 (0.001, 0.002)b	**0.028 (0.025, 0.032)b**
Female-targeted	**All localities combined**	1404	83.8	0.053 (0.050, 0.056)a	**0.045 (0.042, 0.047)a**	0.009 (0.008, 0.010)a
Male-targeted		1657	5.8	0.063 (0.060, 0.066)b	0.004 (0.001, 0.002)b	**0.059 (0.057, 0.063)b**

Across the 3 localities, total Medfly numbers caught in each trap type were highest in Donnybrook, followed by Manjimup, and comparatively very low in the Perth Hills (Table 1). Trapping rates (FTD) compared between localities were significantly different (in the same order) at *P* < 0.001 (Tukey HSD post hoc test) for trapping rate overall, for male Medflies and for female Medflies.

Male-targeted traps were more specific than female-targeted traps (*P* < 0.001, posterior comparison, Table 1). However, the proportion of females caught in each trap type was similar across localities, even though total fly counts varied widely (Table 1).

**Fig. 3.**
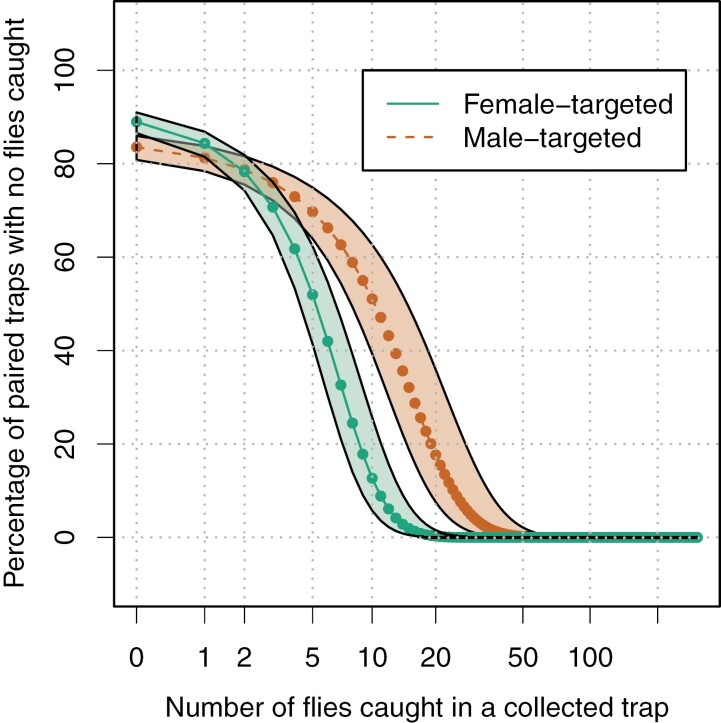
The percentage of traps (with 95% prediction interval) that caught no Medflies in relation to trap catch in the paired trap (male-targeted: dashed line; female-targeted: solid line), as estimated using binomial GLM.

Although total trap catch was similar, female-targeted traps caught at least one Medfly on more occasions (177, 95% bootstrap CI 136-181) than did male-targeted traps (158, 95% bootstrap CI 154-200). Male-targeted traps were more likely to catch no flies if their paired female-targeted trap caught zero or one fly (*P* < 0.05, posterior comparison, solid line, Fig. 3) than the converse (dashed line, Fig. 3). However, female-targeted traps were less likely to catch any Medflies when their paired male-targeted trap caught 4 or more Medflies (*P* < 0.05, posterior comparison, dashed line, Fig. 3) than the converse (solid line, Fig. 3). In the extreme case, if a female-targeted trap caught at least 20 Medflies, it almost guaranteed that a male-targeted trap would trap at least one fly (green line, Fig. 3); conversely, a male-targeted trap needed to trap at least 50 Medflies to similarly guarantee that a female-targeted trap caught any Medflies (orange line, Fig. 3).

### Changes in Monthly Trap Captures

**Fig. 4.**
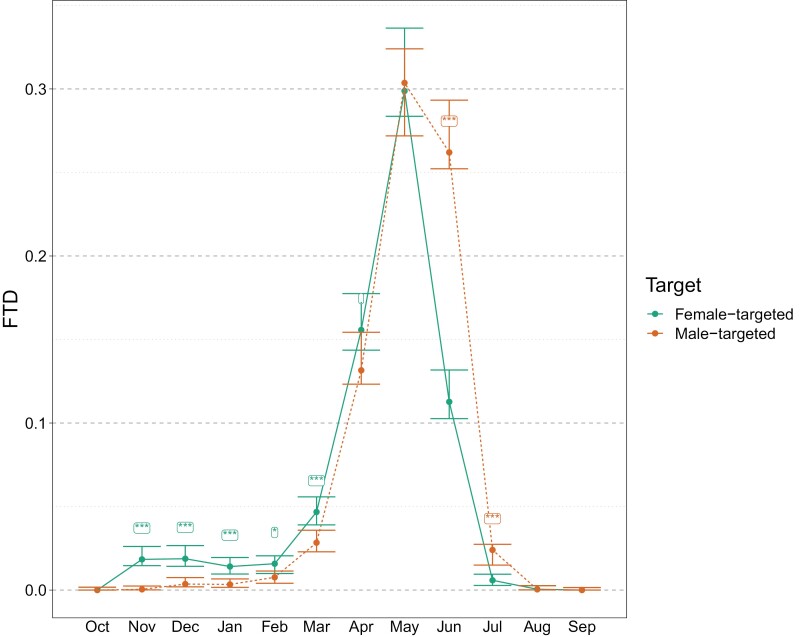
Mean monthly Medflies per trap per day (FTD) caught medflies from female-targeted and male-targeted traps, pooled across the 3 localities and 2 years. Bayesian 95% credible intervals are given, as well as significance between trap targets (* denotes < 0.05, ** < 0.01, and ***<0.001).

Overall, FTD was very low to zero through to Feb before increasing to a peak in May and declining again to very low by Jul to Aug (Figure 4). This pattern was similar in both female- and male-targeted traps, although trap catches increased earlier and faster in female-targeted traps (*P* < 0.05, posterior comparison, Nov to Apr) and remained higher in male traps in June and July.

**Fig. 5.**
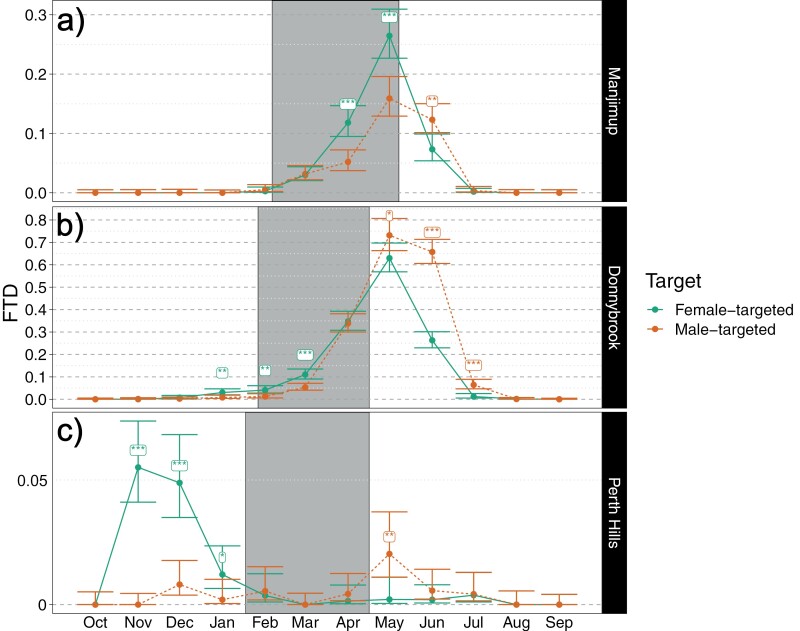
Mean monthly Medflies per trap per day (FTD) in female-targeted traps and male-targeted traps at the 3 localities, averaged across the 2 years. Gray shading indicates the timing of the harvest season of apples at each locality. Bayesian 95% credible intervals are given, as well as significance between trap targets (* denotes < 0.05, ** < 0.01, and ***<0.001). Note the different scales on the *y*-axis for each locality; consistent horizontal grid lines with 0.05 FTD spacing are given to enable comparisons of scale across localities.

Peak seasonal FTD was highest in Donnybrook and very low in the Perth Hills (Fig. 5). Phenology of Medflies was similar in Manjimup and Donnybrook, with FTD remaining very low through to the start of the apple harvest and peaking toward the end of harvest (Manjimup) or soon after harvest completion (Donnybrook). Female-targeted traps caught more Medflies than male-targeted traps when trap catch started to increase (*P* < 0.05, posterior comparison, Donnybrook) and during the last months of apple harvest (P < 0.05, posterior comparison, Manjimup). In contrast, FTD was higher in male-targeted traps than female-targeted traps after harvest had ended, with the difference being most pronounced in June in Donnybrook. A very different phenology was observed in the Perth Hills, although peak FTD was also much lower (Fig. 5). Here FTD peaked well before apple harvest commenced (Nov—Jan) and was near zero through to end harvest period. Interestingly, almost all Medflies caught prior to harvest were in female-targeted traps, and there was a small peak in Medflies caught in male-targeted traps immediately after harvest (May).

### Sex Ratio

**Fig. 6.**
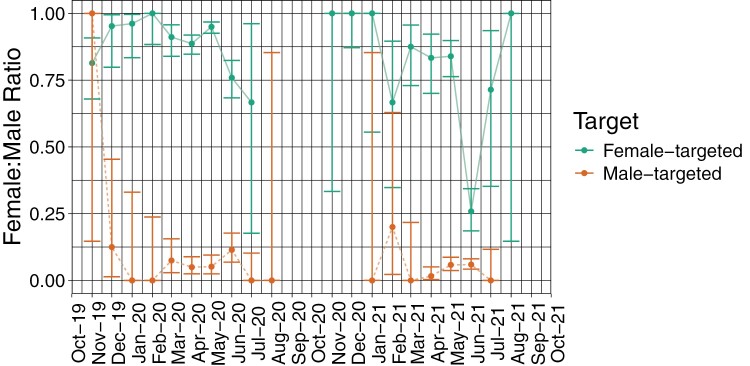
Total sex ratio (F:M) caught in male and female-targeted traps across the 2 years. No Medflies were caught in Sep or Oct in either season. Error bars show the Jeffreys 95% confidence interval for the binomial proportions.

Overall, male-targeted traps had higher specificity (94.2% males) than female-targeted traps (83.8% females) (*z* = 34.70, *P* < 0.001) (Table 1). There was a year-effect for female-targeted traps, which had lower specificity in year 2 (*z* = 10.24, *P* < 0.001) (Fig. 6). There was also a seasonal effect on sex-ratios in both trap-types (Fig. 6). Male-targeted traps caught a higher proportion of females than expected early in the season (*P* < 0.001, Tukey HSD post hoc test, Nov–Dec) when Medfly trap captures were rare, whereas female-targeted traps caught a higher proportion of males late in the season (*P* < 0.001, Tukey HSD post hoc test, Jun–Jul).

## Discussion

Female- and male-targeted traps are used to monitor Medfly populations to inform management decisions worldwide, but there is no clear guidance on which trap type should be used and when. We found that both trap types performed similarly overall, although there were important seasonal and locality effects. Female-targeted traps caught more Medfly earlier in the apple production season (when trap catch was increasing), and male-targeted traps later in the season (when trap catch was declining). This was consistent across all 3 localities. Female-targeted traps were also more likely to capture a single Medfly when their paired male-targeted trap caught no Medflies and capture no Medflies when their paired male-targeted trap caught many. Together, results suggest that either or both trap-types could be used for monitoring Medfly populations, with the optimal solution being dependent on the intended application.

The value of a surveillance trap is determined by both the proportion of the target population that is in a trappable state (attracted to the lure) and the attractiveness strength/range of the lure, both of which can vary with local conditions and season (Weldon et al. 2014, Bau and Cardé 2016). This in turn can determine the surveillance design: for example, low attractiveness can be compensated by increased trapping density (van Klinken et al. 2023) and trap catch thresholds for management can be calibrated to trap sensitivity (Lance 2014, Suckling et al. 2015, Drummond and Collins 2020). Although having very different targets within the Medfly population, both tested trap-types caught largely similar FTD during the harvest period, when fruit are the most susceptible to Medfly. This is consistent with a previous study in Western Australia that was conducted in stone and pome orchards (Broughton and De Lima 2002, Broughton et al. 2015). However, we found important seasonal differences between traps, that were particularly critical when trap catch was very low (Perth Hills). As discussed below, these differences may reflect both changes in the proportion of the population that is trappable, and differences between sexes in seasonal dispersal behavior.

Female-targeted traps had moderately high specificity (85% overall). As female-targeted traps are food-based it is not unusual to capture males who also seek protein for development (Epsky et al. 2014). These results concurred with 2 other Australian Studies: 90–93% (Broughton and Rahman 2017), and 76.1–95.6% (Broughton and De Lima 2002); and one in Greece: 77.0–85.7% (Papadopoulos et al. 2001). Nonetheless, lower specificity has also been reported: 50.8–89.4% (Epsky et al. 1999); 62.8–66.7% (Navarro-Llopis et al. 2008) and 48.4–59.6% (Sciarretta et al. 2018). However, trap type, trap duration, trapping density, season, re-luring frequency, and host type were variable between studies, which may have affected performance. In our study, specificity decreased late in the season (June, especially in year 2), perhaps reflecting late-season male dispersal (see below).

Female-targeted traps consistently caught more Medflies earlier in the apple season, relative to male-targeted traps. This is consistent with female Medfly lures being food-based attractants (Epsky et al. 2014) that are more attractive to virgin females that are seeking protein prior to host maturation than females that are already sexually mature (FAO, 2018, Sciarretta et al. 2018). Although this pattern was consistent across the 3 localities the timing differed, with more females caught in Nov to Jan in the Perth Hills (1-3 months prior to harvest), Jan to Mar in Donnybrook (early harvest), and Apr to May in Manjimup (mid-late harvest). Higher trap catch in female-targeted traps was not, however, consistent across all studies. Papadopoulos et al. (2001) did find that female-targeted traps caught a larger proportion of Medflies 4–6 weeks earlier than male-targeted traps in a mixed orchard in Thessaloniki Greece. However, Manrakhan et al. (2017) found that male-targeted traps consistently caught similar or more medflies than female-targeted traps over a 14 month period in commercial citrus orchards in South Africa, and caught medflies earlier in the season than female-targeted traps but, trap catch was also high through most months in their study. Results from comparative trapping across 30 months on 2 commercial citrus orchards 72 km north of our Perth Hill study sites were inconsistent (Broughton and de Lima 2002), with male-attractant traps mostly outperforming female-attractant traps early in the season in one orchard in one year, and the reverse occurring at the other orchard in the same year. Again, in contrast to our study, the male-attractant traps outperformed female-attractant traps by over 2 to 1.

In our study, male-targeted traps were highly specific capturing 94% males. This is consistent with previous studies. Epsky et al. (1999) found Jackson traps baited with trimedlure caught males 80.9–100% of the time. Papadopoulos et al. 2001 found Jackson traps baited with trimedlure captured males 100% of the time, and Broughton and De Lima (2002) found Lynfield traps baited with Capilure trapped males 92.1–100% of the time. Sciarretta et al. (2018) found Jackson traps baited with trimedlure caught males almost 100% of the time, with the occasional female but these were not reported on.

Male-targeted traps caught more females when trap catches in female-targeted traps peaked. All male-targeted Medfly traps utilize male-attractant para-pheromones (Burk and Calkins 1983, Sivinski and Calkins 1986). However, male-targeted para-pheromones such as trimedlure can be attractive to females if they are unmated and sexually mature, at the time of day when mating usually occurs, or when the population of wild males is low (Fitt 1981, Burk and Calkins 1983). This suggests that the females may be responding to trimedlure as it could increase their probability of finding a mate. The higher female-male sex ratio we observed early in the season in male-targeted traps may be attributed to female Medflies being unmated at that time. Alternatively, females may have been more abundant or active then.

Male-targeted traps consistently caught more Medflies than female-targeted traps later in the season (Jun to Jul) in all localities. This coincided with the end of the apple season, with few Medflies caught in Jul, soon after the onset of winter. Broughton et al. (2015) suggest that during this period, pome trees lose their leaves and male Medfly begin to disperse in search of suitable over-wintering sites. The simultaneous increase in trap catch in male-targeted traps and increase in the proportion of males caught in female-targeted traps in our study supports this hypothesis and appears to be a result of increased dispersal (and therefore trap-encounter rate) rather than an increase in trap attractiveness or proportion of males that are ‘trappable’.

Our trapping documented a very different Medfly phenology in the Perth Hills when compared to Manjimup and Donnybrook. Peak abundance in the Perth Hills was 2–3 months prior to harvest, rather than late-apple season or post-harvest. The Perth Hills has a warmer climate, allowing for medflies to develop and build up populations earlier. However, trap catch was very low in our Perth Hills study sites when compared to our other 2 localities, and to previous studies in the locality that found Medfly populations often exceeding 1 FTD in summer and autumn (Broughton et al. 2015). It suggests that the Medfly populations on our study sites were well-managed. Importantly, the female-targeted traps were highly effective at detecting very low levels of Medfly activity early in the season when male-targeted traps were detecting few or no Medflies.

Our results suggest that although female- or male-targeted traps had comparative strengths, both traps would perform similarly if the aim was to detect moderate densities of flies during the harvest period of apples. Female-targeted traps may be superior if the focus is on early detection of Medfly in the apple production season in Western Australia, especially when trap catches are very low. Male-targeted traps may be superior for quantifying post-harvest populations and guiding over-wintering management. A combination of female- and male-targeted traps may therefore give the best insight into seasonal Medfly abundance and phenology. This agrees with previous studies (Epsky et al. 1999, Broughton and De Lima, 2002, Tóth et al. 2004, Broughton and Rahman 2017). Results from our Perth Hills sites where trap catches were very low suggest that using a combination of female and male-targeted traps may also be more sensitive at early detection. However, the relative merits and costs of using 2 trap systems versus increasing the trapping density (van Klinken et al. 2023) would need to be considered.

Both female- and male-targeted traps used in this trial are currently relatively equitable in their setup and ongoing maintenance costs, with female-targeted traps being cheaper to acquire but slightly more expensive to re-lure and male-targeted traps being more expensive to acquire but with cheaper re-luring costs. Female-targeted traps (Biotrap V2 X) cost $4.95AUD with an ongoing cost of $5.72 AUD every 3 months for the replacement of lures and DDVP cubes. Male-targeted traps (Lynfield) cost $11.45AUD with an ongoing cost of $4.22AUD every 3 months for the replacement of lures and DDVP cubes, prices sourced from Biotrap Australia.

Combining female- and male-targeted lures has been considered but was found to reduce the likelihood of detection (Broughton and De Lima 2002, Tóth et al. 2004).

Trap selection will depend on the surveillance goals. In our study system, the deployment of female-targeted traps will be more useful to detect Medfly in the early stages of an apple production season, whereas male-targeted traps are more useful to detect Medfly populations post-harvest. Where practical, the use of both female- and male-targeted traps in combination allows for an accurate picture of both male and female Medfly populations.
